# Cardiac condition during cooling and rewarming periods of therapeutic hypothermia after cardiopulmonary resuscitation

**DOI:** 10.1186/1471-2253-14-78

**Published:** 2014-09-18

**Authors:** Serdar Demirgan, Kerem Erkalp, M Salih Sevdi, Meltem Turkay Aydogmus, Numan Kutbay, Aydin Firincioglu, Ali Ozalp, Aysin Alagol

**Affiliations:** 1 Department of Anesthesiology and Reanimation, Bagcilar Educational and Training Hospital, Şenlikköy Mah, İncir Sokak, No:1/3, Sarı Konaklar Sitesi, B-Blok, Daire:6, Florya/ Bakırköy, Istanbul, Turkey

**Keywords:** Cardiac arrest, Therapeutic hypothermia, Cardiac function, Cardioprotection, Cardiac measurement

## Abstract

**Background:**

Hypothermia has been used in cardiac surgery for many years for neuroprotection. Mild hypothermia (MH) [body temperature (BT) kept at 32–35°C] has been shown to reduce both mortality and poor neurological outcome in patients after cardiopulmonary resuscitation (CPR). This study investigated whether patients who were expected to benefit neurologically from therapeutic hypothermia (TH) also had improved cardiac function.

**Methods:**

The study included 30 patients who developed in-hospital cardiac arrest between September 17, 2012, and September 20, 2013, and had return of spontaneous circulation (ROSC) following successful CPR. Patient BTs were cooled to 33°C using intravascular heat change. Basal BT, systolic artery pressure (SAP), diastolic artery pressure (DAP), mean arterial pressure (MAP), heart rate, central venous pressure, cardiac output (CO), cardiac index (CI), global end-diastolic volume index (GEDI), extravascular lung water index (ELWI), and systemic vascular resistance index (SVRI) were measured at 36°C, 35°C, 34°C and 33°C during cooling. BT was held at 33°C for 24 hours prior to rewarming. Rewarming was conducted 0.25°C/h. During rewarming, measurements were repeated at 33°C, 34°C, 35°C and 36°C. A final measurement was performed once patients spontaneously returned to basal BT. We compared cooling and rewarming cardiac measurements at the same BTs.

**Results:**

SAP values during rewarming (34°C, 35°C and 36°C) were lower than during cooling (P < 0.05). DAP values during rewarming (basal temperature, 34°C, 35°C and 36°C) were lower than during cooling. MAP values during rewarming (34°C, 35°C and 36°C) were lower than during cooling (P < 0.05). CO and CI values were higher during rewarming than during cooling. GEDI and ELWI did not differ during cooling and rewarming. SVRI values during rewarming (34°C, 35°C, 36°C and basal temperature) were lower than during cooling (P < 0.05).

**Conclusions:**

To our knowledge, this is the first study comparing cardiac function at the same BTs during cooling and rewarming. In patients experiencing ROSC following CPR, TH may improve cardiac function and promote favorable neurological outcomes.

## Background

Hypothermia has been used in cardiac surgery for many years for neuroprotection [1,2]. Mild hypothermia (body temperature (BT) maintained at 32–35°C) has been shown to reduce mortality and improve neurological status by 24–30% in patients who have undergone cardiopulmonary resuscitation (CPR) [3]. Although severe hypothermia (BT <31°C) damages cardiac function, mild hypothermia produces a cardioprotective effect [4,5].

Post-cardiac arrest brain injury results from global brain ischemia during the reduction in general blood flow [6], and prolonged cardiovascular collapse negatively impacts neurologic outcome [7]. Therapeutic hypothermia (TH) is the process whereby BT is reduced to a target temperature of 32–34°C and maintained at this temperature for 24–48 hours following cardiac arrest (CA) and return of spontaneous circulation (ROSC) [8-11]. The 2010 International Consensus on CPR and Emergency Cardiovascular Care Science treatment recommendations [12], the 2010 American Heart Association CPR guidelines [13], the 2010 Japan Resuscitation Council Guidelines [14], and the 2010 European Resuscitation Council (ERC) Guidelines [15] recommend TH following ROSC regardless of cardiac rhythm.

The aim of this study was to determine whether patients who were likely to benefit neurologically from TH would also experience favorable effects on cardiac function. Thus, we measured cardiac function during cooling and rewarming periods in patients receiving TH after CPR.

## Methods

**Figure 1:**
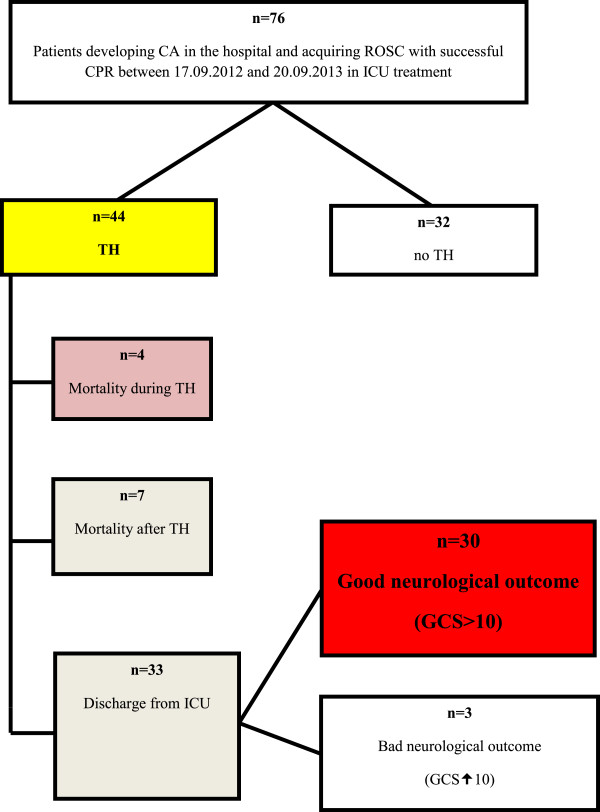
Flow diagram of the study.

Patients were eligible if they developed CA in our hospital and acquired ROSC following successful CPR between September 17, 2012, and September 20, 2013 (Figure 1).

Patients were excluded if they: developed CA outside the hospital; were < 15 years old; had late stage cancer; demonstrated severe cardiogenic shock; were pregnant; had cerebrovascular disease; demonstrated severe heart disease or pulmonary artery thromboembolism; or had aortic dissection or aneurysm.

Participants were admitted to the intensive care unit and received care consistent with 2010 ERC guidelines [15]. For internal BT measurements, a 16 F caliber, 44 cm heat probe silicone urinary catheter (Rusch® sensor series 400, Teleflex Medical IDA Business and Technology Park, Athlone, Ireland) was inserted. A 7 F three lumen central venous catheter (GE-CVC3720Y, GEMED®, Istanbul, Turkey) was placed in the right subclavian vein. A 9.3 F caliber, five-lumen intravascular heat change catheter (IC-3893 AE, ICY Intravascular Heat Exchange Catheter Kit®, ZOLL Circulation Inc., Sunnyvale, CA, USA) was placed into the right femoral vein. A 4 F caliber, 16 cm Philips Continuous Cardiac Output arterial thermodilution catheter (PiCCO Plus®, Pulsion Medical Systems AG, Munich, Germany) was placed in the right femoral artery and connected to the monitor.

Patients were sedated with midazolam 0.02 mg/kg/h and remifentanil 60 μg/kg/h intravenous infusions according to the Ramsay Sedation Scale level 3–4 [16]. Sedation was continued throughout TH and rewarming. During cooling, patients who demonstrated shaking that could adversely affect hemodynamic balance received 0.03–0.05 mg/kg vecuronium intravenously. Consistent with hospital protocol, patients whose mean arterial pressure (MAP) was less than 60 mmHg and unresponsive to a volume challenge received intravenous dopamine and noradrenalin infusion.

Patient BTs were cooled to 33°C using intravascular heat change at a rate of 0.5°C/h (Thermogard XP®, Alsius Corp., Chelmsford, MA, USA). Basal BT, systolic artery pressure (SAP), diastolic artery pressure (DAP), MAP, heart rate (HR), central venous pressure (CVP), cardiac output (CO), cardiac index (CI), global end-diastolic volume index (GEDI), extravascular lung water index (ELWI) and systemic vascular resistance index (SVRI) values were measured at 36°C, 35°C, 34°C and 33°C during cooling. During the measurements, 15 mL of 0.9% saline solution at a temperature of less than 8°C was administered through a central venous catheter over 10 seconds or less. Measurements were obtained with the help of thermodilution curves using the PiCCO® plus device. To ensure reliability, three injections and three series of measurements were performed for each temperature point, and mean values were used for analysis. Incompatible values among injections were deleted and the measurements were repeated. Central catheters were recessed during measurements. BT was held at 33°C for 24 hours prior to rewarming. Rewarming was conducted at 0.25°C/h. During rewarming, measurements were repeated at 33°C, 34°C, 35°C and 36°C. A final measurement was performed when patients spontaneously returned to basal BT.

We compared cooling and rewarming cardiac measurements conducted at the same BTs using means, standard deviations, ranges, medians, ratios and frequencies. Variable distribution was controlled using the Kolmogorov Smirnov test. Repeated measurements were analyzed with paired sample *t*-tests. SPSS 21.0 software (IBM® Corp., Armonk, NY, USA) was used to analyze the data.

Permission to conduct this study was obtained from the Clinical Studies Local Ethics Committee of the Republic of Turkey Ministry of Health, Bagcilar Training and Research Hospital (17.09.2012-76). Written informed consent was obtained from the patients’ first degree relatives.

## Results

Patient demographic characteristics are presented in Table 1. Cardiac measurements during cooling and rewarming are shown in Table 2. SAP measurements during rewarming at 34°C and 35°C were lower than during rewarming at 33°C (P < 0.05). DAP values did not differ during cooling or rewarming. MAP values during rewarming at 34°C, 35°C and 36°C were lower than at 33°C (P < 0.05). HR values during cooling at 35°C, 34°C and 33°C were lower than at basal temperature (P < 0.05). HR values during rewarming at 34°C, 35°C, 36°C and basal temperature were higher than at 33°C (P < 0.05). CVP values during rewarming at 35°C and 36°C were lower than at 33°C (P < 0.05). CO values during cooling at 35°C and 33°C were lower than at basal temperature (P < 0.05). CO values during rewarming to basal temperature were higher than at 33°C (P < 0.05). CI values during cooling at 36°C, 35°C and 33°C were lower than at basal temperature (P < 0.05). CI values during rewarming to basal temperature were higher than at 33°C (P < 0.05). GEDI values during cooling at 36°C were lower than at basal temperature. GEDI values at 33°C were higher than at basal temperature (P < 0.05). GEDI values during rewarming at 35°C and basal temperature were higher than at 33°C (P < 0.05). ELWI values during cooling at 36°C and 35°C were higher than at basal temperature (P < 0.05). SVRI values during cooling at 35°C and 34°C were higher than at basal temperature (P < 0.05). SVRI values during rewarming at 34°C, 35°C, 36°C and at basal temperature were higher than at 33°C (P < 0.05).

**Table 1:** Summary of demographic and clinical characteristics of the 30 patients with cardiac arrest

Age in years (mean ± SD)	49.07 ± 18.04	
Males	17 (56.7%)	
Height (cm)	171.4 ± 6.9	
Weight (kg)	86.3 ± 17.6	
Initial GCS level		
III	23 (76.7%)	
IV	5 (16.7%)	
V	2 (6.7%)	
Cardiac arrest rhythm		
Ventricular tachycardia	7 (23.3%)	
Ventricular fibrillation	18 (60%)	
Asystole	5 (16.7%)	
CPR duration (min)	20 ± 9.4	
Coronary angiography	Yes	9 (30.0%)
	None	21 (70.0%)
Time from ROSC to start of TH induction (min)	46.86 ± 14.73	
SOFA Scores	11.93 ± 1.23	
APACHE II Scores	22.77 ± 4.45	

GCS: Glasgow Coma Scale.

CPR: Cardiopulmonary Resusication.

ROSC: Return of Spontaneous Circulation.

TH: Therapeutic Hypothermia.

SOFA: Sepsis-related Organ Failure Assessment.

APACHE: Acute Physiology and Chronic Health Evaluation.

**Table 2:** Variation for the cardiac thermodilution maesurements at the same body temperature during cooling and rewarming periods

**Cooling (C)/Rewarming (RW) periods**		**Mean. ± s.d.**	**p**		**Mean. ± s.d.**	**p**
**BTbasal-C/BTbasal-RW**	**SAP**	10,7 ± 33,8	0,094	**SVRI**	792 ± 672	***0,000***
**BT36-C/BT36-RW**		12,9 ± 29,5	***0,024***		1089 ± 1791	***0,002***
**BT35-C/BT35-RW**		14,8 ± 29,1	***0,009***		1178 ± 1444	***0,000***
**BT34-C/BT34-RW**		11,9 ± 29,9	***0,038***		777 ± 1094	***0,001***
**BT33-C/BT33-RW**		−6,7 ± 30,7	0,242		72 ± 1325	0,768
**BTbasal-C/BTbasal-RW**	**DAP**	8,2 ± 19,7	***0,030***	**CO**	−1,1 ± 1,6	***0,001***
**BT36-C/BT36-RW**		10,7 ± 18,3	***0,003***		−0,8 ± 1,6	***0,009***
**BT35-C/BT35-RW**		11,0 ± 19,6	***0,005***		−1,1 ± 1,6	***0,000***
**BT34-C/BT34-RW**		8,1 ± 15,7	***0,008***		−0,8 ± 1,4	***0,003***
**BT33-C/BT33-RW**		2,2 ± 19,2	0,529		−1,0 ± 1,5	***0,002***
**BTbasal-C/BTbasal-RW**	**MAP**	6,7 ± 24,4	0,141	**CI**	−0,6 ± 1,0	***0,002***
**BT36-C/BT36-RW**		11,8 ± 19,9	***0,003***		−0,5 ± 0,8	***0,001***
**BT35-C/BT35-RW**		12,0 ± 19,8	***0,002***		−0,7 ± 0,9	***0,000***
**BT34-C/BT34-RW**		9,1 ± 19,7	***0,017***		−0,5 ± 0,8	***0,002***
**BT33-C/BT33-RW**		−2,3 ± 24,2	0,602		−0,5 ± 0,9	***0,012***
**BTbasal-C/BTbasal-RW**	**HR**	4,1 ± 19,9	0,273	**GEDI**	22,0 ± 140,2	0,397
**BT36-C/BT36-RW**		3,8 ± 24,2	0,397		−72,3 ± 167,1	***0,025***
**BT35-C/BT35-RW**		−3,4 ± 18,5	0,326		−83,8 ± 285,3	0,118
**BT34-C/BT34-RW**		−2,7 ± 16,5	0,384		−18,6 ± 171,8	0,557
**BT33-C/BT33-RW**		2,7 ± 19,5	0,450		−0,9 ± 117,5	0,966
**BTbasal-C/BTbasal-RW**	**CVP**	0,8 ± 7,2	0,562	**ELWI**	0,1 ± 2,9	0,902
**BT36-C/BT36-RW**		1,8 ± 5,4	0,074		1,3 ± 4,1	0,083
**BT35-C/BT35-RW**		1,8 ± 5,3	0,081		1,0 ± 3,8	0,148
**BT34-C/BT34-RW**		1,9 ± 5,6	0,078		0,6 ± 3,6	0,394
**BT33-C/BT33-RW**		1,7 ± 5,6	0,111		0,7 ± 2,8	0,206

Paired sample t test.

***SAP*** measurements did not demonstrate any difference at Tbasal-C/Tbasal-RW and 33°C (p > 0.05). SAP values during rewarming at 34°C, 35°C, 36°C were lower as compared to measurements in cooling period (p < 0.05). ***DAP*** measurements did not demonstrate any difference at 33°C (p > 0.05). DAP values during rewarming at Tbasal-RW, 34°C, 35°C, 36°C were lower as compared to the measurements during cooling period. ***MAP*** meaurements did not demonstrate any difference at Tbasal-C/Tbasal-RW and 33°C (p < 0.05). MAP values during rewarming at 34°C, 35°C, 36°C were lower as compared to measurements during cooling period (p < 0.05). ***HR*** and ***CVP*** measurement values at the same body temperature during rewarming and cooling period did nor demonstrate any difference (p > 0.05). ***CO*** measurements were higher during the rewarming period (p < 0.05). ***CI*** measurements were higher during the rewarming period (p < 0.05). ***GEDI*** and ***ELWI*** measurements during rewarming and cooling periods did not demonstrate any difference at the same body temperatures (p > 0.05). ***SVRI*** measurements during rewarming and cooling periods at 33°C did nor demonstrate any difference (p > 0.05). SVRI values during rewarming period at 34°C, 35°C, 36°C and Tbasal-RW period were lower (p < 0.05).

C: Cooling, RW: Rewarning, BT: Body temperature, SAP: Systolic arterial pressure, DAP: Diastolic arterial pressure, MAP: Mean arterial pressure, HR: Heart rate, CVP: Central venous pressure, SVRI: Systemic vascular resistance index, CO: Cardiac output, CI: Cardiac index, GEDI: Global end-diastolic volume index, ELWI: Extravascular lung water index.

Some cardiac function values differed when measured at the same temperature during cooling and rewarming phases. SAP values at 34°C, 35°C and 36°C were lower during rewarming compared with cooling (P < 0.05). DAP values at basal temperature, 34°C, 35°C and 36°C were lower during rewarming than during cooling. MAP values at 34°C, 35°C and 36°C were lower during rewarming than during cooling (P < 0.05). HR, CVP, GEDI and ELWI values at the same BT did not differ between cooling and rewarming periods. CO values were higher during rewarming than during cooling (P < 0.05). CI values were higher during rewarming than during cooling (P < 0.05). SVRI values at 34°C, 35°C, 36°C and basal temperature were lower during rewarming than during cooling (P < 0.05) (Table 2).

## Discussion

In our comparison of cardiac function during the cooling and rewarming following CA and ROSC, we found that the greatest improvement occurred at 33°C at the beginning of the rewarming period and that improvement in cardiac function continued throughout the rewarming period. This was consistent with a recent study showing that patients kept at 33°C BT for about 24 hours experienced improved cardiac function during rewarming.

The rationale for applying TH in patients experiencing ROSC after CPR is to improve brain tolerance to ischemia. There are increasing reports of neurological disease-free survival and recovery in these situations [17,18]. Experimental studies demonstrated that hypothermic blood introduced to the coronary sinus during heart ischemia minimizes reperfusion damage and infarct area [19]. TH also protects contractility, prevents microvascular obstructions, and reduces left ventricular remodeling [20]. In addition, the reduced metabolic rate, decreased tissue apoptosis, and heat shock protein induction offer cellular protective effects [21]. The cardioprotective effects of TH are directly related to timing. Late induction or a slow cooling speed does not improve cardiac function [22,23]. The time from ROSC to TH induction was about 45 minutes in our study. Although we selected 0.5°C/h for cooling, new studies suggest that faster cooling (>0.5°C/h) to the targeted BT is associated with better cardioprotection [24,25]. According to Nagao et al., TH is more effective if begun during CPR prior to ROSC, and early cooling during CA is neuroprotective and myocardioprotective [26]. TH during CPR also increases resuscitation rates, improves cardiac and left ventricular function and decreases the myocardial infarct area [27,28].

Schmidt-Schweda et al. showed that HR decreased whereas stroke volume (SV), CI and CO increased in cardiomyopathic patients receiving short-term cooling [29]. However, these improvements were lost during rewarming, and the authors interpreted TH application as having a positive inotropic effect [29]. In contrast, CO and CI in our study improved at the end of the cooling period and throughout rewarming, with the highest levels occurring at the end of the process. As per our protocol, dopamine and noradrenalin infusions were decreased or eliminated during rewarming. There were significant increases in CO and CI, and a significant decrease in SVRI, but no significant change in GEDI or HR during the rewarming period compared with the cooling period. This might imply that there was an increased SV during the rewarming period resulting from either increased contractility or decreased afterload or both. Unfortunately, recent study findings could not distinguished between the two possibilities. Gibson et al. suggested that the TH adverse effects of bradycardia and hypotension actually benefit patients, similar to the effect of beta-blockers [30]. When Riaz et al. reported results of cooling patients to 32°C to 34°C for 24 hours following ventricular tachycardia or ventricular fibrillation, they emphasized the risk of QT interval prolongation, particularly in those who had received amiodarone [31].

Management of patients receiving CPR during TH is difficult [8,9]. The most frequent cause of complex hemodynamic instability is arterial hypotension, which is characterized by hypovolemia, reversible myocardial stunning and excessive vasodilation [32-36]. Pulmonary dysfunction is related to cardiogenic pulmonary edema caused by left ventricular dysfunction and non-cardiogenic edema caused by inflammatory, infective and physical damage [9]. We used PiCCO® monitoring to improve patient care and track cardiac function during this difficult post-ROSC period. It has been reported that PiCCO® monitoring with transpulmonary thermodilution may be compromised in patients receiving TH [37,38]. For this reason, we compared cardiac function measurements at the same BTs during cooling and rewarming. However, there are inadequate prospective studies published regarding the reliability of PiCCO® measurements at low BTs. Further prospective studies similar to ours are needed. Fluid and electrolyte balance during TH is an important challenge [39]. Cooling can cause peripheral vasoconstriction, reduce vascular volume and CVP, and the patient seems relatively hypovolemic [40]. In our study, we detected no significant change in CVP and HR, but we did detect higher CO and CI values. During these therapeutic cooling and rewarming periods, patient fluid status must be monitored closely to maintain volume balance, and a vasopressor added when necessary to maintain adequate blood pressure.

Post-resuscitation hemodynamic instability is characterized by a low CI and normal or low filling pressures [33]. The CI rapidly increases 24 hour after CA, independent of filling pressures and vasoactive agents [41]. Recovery of this myocardial dysfunction is most often obtained within 24–48 hours and this condition might have played a role in our findings [33]. Full recovery with discontinuation of inotropic support has usually occurred by 72 hours after ROSC [41]. In contrast, TH has been shown to produce negative effects as well as cardioprotective effects. Espinoza et al. showed that TH in pigs decreased SV, ejection fraction and strain in hearts paced at 33°C, and concluded that TH may lead to systolic and diastolic dysfunction [42]. When Bassin et al. induced hypothermia in sheep using venous extracorporeal circulation, they observed increased heterogeneity in hypothermic depolarization and repolarization during cardiac rhythm tracking by 12-derivation ECG, thus providing strong evidence that hypothermia to 34°C produced adverse effects [43].

Several limitations of the current study deserve mention. We did not select a control group so we do not know how cardiac functions would have differed from patients who did not receive TH. We were also unable to study patients undergoing angioplasty following CA as a result of myocardial infarction or patients with known underlying cardiac disease. Once TH was complete, we did not collect PiCCO® measurements. Thus, we could not determine whether the beneficial cardiac function changes persisted over time.

## Conclusions

This study found that CO and CI increased together during rewarming following TH. The lack of difference in HR measurements indicate that CO and CI increased by increases in TH and SV. To our knowledge, this is the first study comparing cardiac function at the same BTs during cooling and rewarming. In patients experiencing ROSC following CPR, TH may improve cardiac function. Further studies are needed to confirm these results and the relationship between TH and post-resuscitation myocardial function in patients with different neurological status.

## Abbreviations

MH: Mild hypothermia; BT: Body temperature; CPR: Cardiopulmonary resuscitation; TH: Therapeutic hypothermia; ROSC: Return of spontaneous circulation; SAP: Systolic artery pressure; DAP: Diastolic artery pressure; MAP: Mean arterial pressure; CO: Cardiac output; CI: Cardiac index; GEDI: Global end-diastolic volume index; ELWI: Extravascular lung water index; SVRI: Systemic vascular resistance index; CA: Cardiac arrest; ERC: European Resuscitation Council; HR: Heart rate; CVP: Central venous pressure; SV: Stroke volume.

## Competing interests

The authors declare they have no competing interests.

## Authors’ contributions

SD conceived and designed the study, conducted the literature review, wrote and critically reviewed the paper. KE conceived and designed the study, analyzed and interpreted the data, conducted the literature review, wrote and critically reviewed the paper. MSS supervised the study, conducted the literature review, wrote and critically reviewed the paper. MTA supervised the study, analyzed and interpreted the data. AA supervised the study, analyzed and interpreted the data, and critically reviewed the paper. NK provided funding and materials, and collected and processed the data. AF provided funding and materials, and collected and processed the data. AO provided funding and materials. All authors read and approved the final manuscript.

## Pre-publication history

The pre-publication history for this paper can be accessed here:

http://www.biomedcentral.com/1471-2253/14/78/prepub
